# Analyzing long-term impacts of ungulate herbivory on forest-recruitment dynamics at community and species level contrasting tree densities versus maximum heights

**DOI:** 10.1038/s41598-020-76843-3

**Published:** 2020-11-20

**Authors:** Ursula Nopp-Mayr, Susanne Reimoser, Friedrich Reimoser, Frederik Sachser, Leopold Obermair, Georg Gratzer

**Affiliations:** 1 Department of Integrative Biology and Biodiversity Research, Institute of Wildlife Biology and Game Management, University of Natural Resources and Life Sciences, Vienna, Gregor-Mendel-Straße 33, 1180 Vienna, Austria; 2 Department of Integrative Biology and Evolution, Research Institute of Wildlife Ecology, University of Veterinary Medicine, Savoyenstraße 1, 1160 Vienna, Austria; 3 Hunting Association of Lower Austria, Wickenburggasse 3, 1080 Vienna, Austria; 4 Department of Forest- and Soil Sciences, Institute of Forest Ecology, University of Natural Resources and Life Sciences, Vienna, Peter Jordan-Straße 82, 1190 Vienna, Austria

**Keywords:** Plant ecology, Community ecology, Ecology, Forest ecology

## Abstract

Herbivores are constitutive elements of most terrestrial ecosystems. Understanding effects of herbivory on ecosystem dynamics is thus a major, albeit challenging task in community ecology. Effects of mammals on plant communities are typically explored by comparing plant densities or diversity in exclosure experiments. This might over-estimate long-term herbivore effects at community levels as early life stage mortality is driven by a multitude of factors. Addressing these challenges, we established a set of 100 pairs of ungulate exclosures and unfenced control plots (25 m^2^) in mixed montane forests in the Alps in 1989 covering a forest area of 90 km^2^. Investigations ran until 2013. Analogous to the gap-maker–gap-filler approach, dynamically recording the height of the largest trees per tree species in paired plots with and without exclosures might allow for assessing herbivore impacts on those individuals with a high probability of attaining reproductive stages. We thus tested if recording maximum heights of regenerating trees would better reflect effects of ungulate herbivory on long-term dynamics of tree regeneration than recording of stem density, and if species dominance patterns would shift over time. For quantifying the effects of ungulate herbivory simultaneously at community and species level we used principle response curves (PRC). PRCs yielded traceable results both at community and species level. Trajectories of maximum heights yielded significant results contrary to trajectories of total stem density. Response patterns of tree species were not uniform over time: e.g., both Norway spruce and European larch switched in their response to fencing. Fencing explained about 3% of the variance of maximum tree heights after nine years but increased to about 10% after 24 years thus confirming the importance of long-term surveys. Maximum height dynamics of tree species, addressed in our study, can thus reflect local dominance of tree species via asymmetric plant competition. Such effects, both within and among forest patches, can accrue over time shaping forest structure and composition.

## Introduction

Herbivores are constitutive elements of most terrestrial ecosystems in which the co-evolved interactions between plants and herbivores can vary widely in their intensity and feedbacks^1^. Herbivory is a major driver of dynamics in many ecosystems (e.g.^2–4^) and a major selective pressure leading to numerous evolutionary adaptations^5,6^ like chemical or morphological defence strategies of plants or strong dependencies of herbivores on food availability. In turn, plants impact herbivore survivorship and fecundity in terms of quantity and quality of feeding resources. As a numerical response, enhanced food availability is typically linked with increased consumption rates. Vice versa, herbivory by ungulates can reduce or promote structural diversity and plant species diversity depending on the intensity and selectivity of feeding, the palatability and functional redundancy of plants, and other factors^7–9^. Top-down effects of herbivores depend on (individual) feeding strategies, which include adaptive trade-offs between nutrient intake and avoidance of predation risk^10^. In the past, a multitude of studies on herbivory focused on the fraction of aboveground net primary production consumed by herbivores thereby highlighting the global role of herbivory and tackling questions of top-down versus bottom-up control in ecosystems^10–15^. Bottom-up forces comprise abiotic factors that drive primary production (like soil fertility), whereas top-down forces address impacts of higher trophic levels on lower levels^16^. Such studies usually do not clarify lasting effects of herbivory on dynamics of plant communities^1,17^.

Effects of mammals on plant communities are typically explored by exclosure experiments^18–23^ impeding access of mammalian herbivores to study plots. However, year-round absence of native herbivores does not display natural terrestrial ecosystem conditions. Correspondingly, a conceptual framework of effects of defaunation on different trophic levels within forest communities has been provided in the past, showing long-term effects of defaunation on a pristine tropical forest^24^. Comparing exclosures and coupled open access plots, the consumption of organic matter by herbivores might be expressed as differences in net primary production^25^, in plant densities^8,18,20^, or, at community level, in structural diversity or species diversity^8,26–30^.

Quantifying long-term effects of herbivory on plant species composition and dynamics of ecosystems faces several challenges: plants, and particularly trees, show strong ontogenetic variation in herbivory. This is caused by higher ratios of nutrient-rich (cotyledons, leaves) versus nutrient-poor (stem) plant structures, by reduced defence of early developmental stages after stored carbohydrate reserves in seeds and cotyledons are allocated^31–33^ and by a higher number of herbivore guilds being able to consume small plants. Assessing herbivory thus has to capture these early life stages, especially if consumption rates differ with species. Trees in these life stages, however, are frequently under-dispersed^34^ and show high densities. This leads to density-dependent mortality and is typically reflected in exponentially decreasing stem density distributions, caused by stage specific mortality factors (like adverse microsite conditions^35^, pathogens^36^, competition, or herbivory^37^). Consequently, only a comparatively small number of progenies survive^38^ and only a small portion of seedlings finally grows into the forest canopy and can thus reproduce. Thus, initial herbivore effects at early developmental stages might be accompanied by a multitude of co-occurring or subsequent processes and might not be significant drivers of dynamics at later stages. They are only part of the compensatory mortality dynamics. Accordingly, short time surveys of herbivore effects in terms of reductions in stem numbers or net primary production might over-estimate herbivore effects in the long-term and at community levels, particularly for tree species composition. These problems are well known in studies of tree regeneration in forest gaps where species composition at early stages based on seedling densities can be a misleading proxy of future species composition^39–41^. This is overcome by assessing gap fillers or definite gap fillers (sensu^39^) as those individuals with a high probability for replacing a gap maker. Analogous to this approach, dynamic recording of the height of the highest tree individuals per species in paired plots with and without exclosures over time (thereby allowing for changes in dominance with time) may allow for assessing herbivore impacts on those individuals with a high probability of reaching the main canopy and getting into reproductive stages. Although tree height growth is a fundamental factor in juvenile survivorship (e.g.^42^) in turn determining the chance of reaching reproductive size^41,43^, tree heights are less frequently considered^30^ than stem numbers (per height class^14^) or species abundances (e.g.^18^), when exploring effects of herbivores on forest tree communities^20,30^. When tree heights are used to depict herbivore impacts, growth patterns and growth histories of tagged tree individuals are frequently taken as reference for the quantification of herbivory-mediated reductions in height growth^15,24,44–46^. This approach, however, requires either tagging of individuals in high densities (with all associated challenges of adequate numbers of replications of study sites), or holds the risk of selecting individuals that do not represent the dominant ones that will have a high chance of forming the future canopy.

To compare approaches assessing herbivory effects based on stem density or on heights of highest individuals per plot, we used a unique data set of deer exclosures gained in mixed forest communities (particularly spruce, beech, fir) for up to 24 years^47^. For each tree species, both densities and heights were recorded, which allows for testing the following hypotheses: (1) recording maximum heights of regenerating trees better reflects effects of ungulate herbivory on long-term dynamics of tree regeneration than recording of stem density and (2) species dominance patterns shift over time; thus, results from surveys with short and medium duration differ from long-term results, when individuals have reached later development stages (with terminal heights being no longer accessible for browsing by ungulates as is the case with thickets).

## Methods

### Study area

Regeneration dynamics were monitored on the forest area (90 km^2^) of the mountain massif “Höllengebirge” (highest elevation 1862 m), being part of the northern Limestone Alps in Upper Austria, Central Europe (47°49′ N, 13°30′ E). The forest is located around a central plateau that lays above the forest line. Geological substrates are dominated by limestone and dolomite, typical soils are Rendzinas and relictic loams. The climate of the region is submaritime, with long winter periods and cool, wet and short summer periods. Mean annual temperature of the area is about 6–8 °C (depending on altitude) and mean annual precipitation reaches approx. 1800 mm. Annual precipitation is characterized by a bimodal temporal pattern, with one maximum occurring at July and another one between November and January. The area is typically covered by snow on approx. 170 days per year, with large inter-annual variation^48^. Typical forest communities are montane mixed forests comprising of Norway spruce (*Picea abies*), silver fir (*Abies alba*), European beech (*Fagus sylvatica*), ash (*Fraxinus excelsior*), and sycamore maple (*Acer pseudoplatanus*). They are classified as *Helleboro nigri-Fagetum* and *Adenostylo glabrea-Fagetum*^49^. The silvicultural system is permanent forest (shelterwood and strip-selection cutting) with natural forest regeneration and additionally strip clear-cuts with supplementary afforestation. During the investigation period no serious climatic events (e.g. drought, wind throw, snow break) or insect calamities occurred.

The mean ungulate density on the forested area in spring (without newborn calves, fawns and kids) is in total about 20 animals per km^2^, consisting of approximately four red deer (*Cervus elaphus*, counted at winter-feeding stations), five to ten roe deer (*Capreolus capreolus*, estimated by local hunters), and six to ten chamois (*Rupicapra rupicapra*, estimated by local hunters) per km^2^; the hunting season runs from May to December. Hunting bags were 1 to 2 red deer, 1 to 4 roe deer, and 1 to 2 chamois per km^2^ and year, on a constant level over the observation period.

### Sampling method

We established 100 pairs of ungulate exclosures (excluding the occurring red deer, roe deer and chamois) and unfenced control plots at altitudes between 500–1100 a.s.l. in 1989 and investigated them until 2013. Minimum distance between plot pairs is 200 m. Plots were installed at forest sites where tree regeneration was potentially possible (i.e. mature forests providing seed trees), and where initial stages of regeneration already existed in the majority of cases or were expected to occur in the near future^50,51^. In choosing sites we also considered that light conditions would enable further development of seedlings (maximum canopy density by seed trees about 80%) and that a subsequent dense canopy closure was unlikely. Smaller mammals like hares (*Lepus europaeus*) or herbivore voles and mice were able to enter the exclosures. The type of treatment (fencing vs. control) was randomly attributed with a coin toss. Exclosures were constructed with a 2.0 m tall, galvanized fence outlining an area of 6 × 6 m. Distances between exclosures and corresponding control plots ranged from 5 m up to max. 20 m with largest possible congruence of site and stand conditions between exclosures and control plots. In this alpine environment with very high spatial heterogeneity, larger plots would have inevitably implicated more divergence between fence and control plots even at the beginning of the survey, which would have biased our records. Within the 6 × 6 m area of each fence and control plot, we recorded plant data within the central 5 × 5 m square (i.e. survey area without “edge effects”), which was permanently marked with metal rods. Data were first recorded in the year of establishment of exclosure/control pairs and afterwards in a three-year cycle. Thereby, the height of the tallest tree of all species per sampling plot (25 m^2^, each fenced and unfenced) was recorded using the following height classes: ≤ 10 cm, 11–25 cm, 26–40 cm, 41–70 cm, 71–100 cm, 101–130 cm, 131–160 cm, 161–200 cm, 201–250 cm, 251–300 cm, … (continuing 50 cm classes). Up to the 18-year period, we further recorded stem numbers and height classes of all trees. From the original 100 control samples 82 plots still existed after nine years, 65 after 12 years, 43 after 18 years and 17 plots could be further investigated until a 24-year period. The other plots dropped out mainly due to rockfall or single windthrown trees damaging them.

No animal experiments were carried out. The ungulates were neither caught nor influenced by medication. They were only denied access to small patches within forests by means of a fence. The fencing was carried out in accordance with all relevant guidelines and regulations. The entire sampling design was optimized to capture effects of ungulate herbivory and to keep other driving factors as constant as possible.

### Data analyses

Targeting at *synchronous* analyses of the effects of ungulate herbivory on recruitment dynamics of tree communities and of all recorded tree species, we had to apply a method, which could cope with a large number of potentially occurring tree species (and thus with numerous categories of one input variable). Traditional approaches exploring effects of herbivory on tree recruitment dynamics like LMs, GLMs or GLMMs usually address only a few tree species (in most cases considering the dominant species^15^), or the response of single species within communities^52^ or community specific indices (e.g. diversity indices, species numbers, total seedling density or seedling heights^14,30,52^). In our study, we recorded up to 19 tree species at the fenced and/or unfenced plots, which would not allow for a convergence of traditional LMs, GLMs or GLMMs when considering interaction terms. We thus used principle response curves (PRC^22,53–55^ with a scaling of two) for data analyses, as we aimed to quantify the effects of ungulate herbivory on recruitment, height dynamics and stem density at both community level and at the level of all occurring single tree species. We presumed that PRC is a valuable approach for analysing regeneration dynamics on exclosure-control plots, looking both at total stem density and at stem heights of the highest trees per tree species (and plot). As height growth is strongly correlated with time, we particularly focused on height growth variance originating from treatment differences across time. PRC is a derivative of redundancy analysis (RDA), which uses sampling dates as set of co-variables and the interaction between sampling dates and treatment as set of explanatory variables. In our case, time series of treatment sites (fenced plots) is compared with corresponding control sites (unfenced plots) and effects of a treatment are represented as deviations from a control (= reference). Performing a PRC means variance partitioning^56^, yielding a variance part which origins from differences in time and another part which origins from treatment differences in time. The PRC focuses on effects of treatments factoring out variations between replicate microcosms and differing dates^54^. The first principal component of variance, being explained by the interaction of treatment and time, is displayed on the y-axis, whereas sampling dates are depicted on the x-axis^54^. Additionally, species scores on a second y-axis reflect the affinity of single species to the community-based response curve. This might be interpreted as weight of a species for the response diagram. Species with the same algebraic sign as the overall response curve show a corresponding response to a treatment, species with scores about zero (matching to the reference line) do not respond to the treatment or show distinctly differing response patterns, whereas species with an opposite sign than the community response are negatively correlated to the overall response curve. We used PRC to compare the outcome of two recording methods, i.e. dynamic maximum heights and total density of individuals.

In contrast to previous studies using PRC (e.g.^22,55^), we did not address changes in community composition (expressed in species numbers or coverage), but trajectories of maximum heights of all occurring tree species. As tree heights had been recorded in terms of height classes, we used the mid-point height per height class interval as representative (e.g. 85 cm for the 71–100 cm height class). To address both recruitment and height dynamics, we set the maximum tree heights of all potentially occurring tree species to zero when tree species were absent on single plots or points of time. This allowed us to adequately represent the following circumstances: (1) Tree species might be present on a fenced plot, while being absent on the control plot and vice versa. Without a zero maximum height value, we would have lost the information, that a given tree species is absent and related comparisons of fenced vs. control plots would have been biased. (2) Tree species might not be present during the first records, but might emerge and disappear at later points of time. Again, only zero maximum height values for all absent tree species could adequately represent such patterns. (3) Zero maximum height values allowed us to represent not only effects of herbivory on growth patterns of all potentially occurring tree species, but also on recruitment, which draws a more comprehensive picture of community and species responses to ungulate herbivory than it would have been the case otherwise. Due to the linear character of subsequent height classes, we did not log-transform height data, already matching the assumption of linearity for the PRC^57^. To address hypothesis (1), we also ran PRCs with total tree density. To depict regeneration, emerging after the installation of the fence/control-plots, we both considered stem numbers per hectare including height class 1 (i.e. plantlets < 10 cm) and stem numbers excluding height class 1. We tested the significance of the PRC with Monte Carlo permutations of the microcosms with permutations (n = 1000) of entire time series in partial RDAs. We further ran Monte Carlo permutation tests (n = 1000) for each sampling date, looking at the significance of the treatment per sampling date. As constrained proportions of variance increased with longer lasting survey periods (particularly relevant for maximum heights), we ran two analyses for testing the sensitivity of PRCs to sample size (i.e. the number of plot pairs): We first calculated PRCs with randomly reduced data sets without resampling and we used random sampling with replacement in a second step to draw subsets for the first two surveys (82 and 65 pairs of sampling plots respectively) consisting of 82 (for the first survey only), 65, 43 and 17 pairs of sampling plots with 500 iterations each. We then computed PRC analyses for each of the 3500 resulting subsets and extracted the constrained and conditional proportion of variance for comparison. To contrast results of PRCs roughly against a more traditional approach, we additionally ran LMs (for maximum height values) per tree species for the 18-year records, considering the treatment (fence vs. control), the point of time and their interaction as fixed factors. We used R 3.6.3^58^ and vegan 2.5–4^59^ for our analyses. For convenience of the workflow we further used RStudio 1.1.463^60^ and the following R-packages: dplyr 0.8.0.1^61^, forcats 0.3.0^62^, ggplot2 3.1.0^63^, purrr 0.3.1^64^, readr 1.3.1^65^, stringr 1.4.0^66^, tibble 2.0.1^67^, tidyr 0.8.3^68^, skimr 1.0.5^69^, rstudioapi 0.7^70^, rmarkdown 1.10^71^, knitr 1.20^72^ and hues 0.1^73^.

## Results

### Dynamics of stem densities and maximum heights over time

**Figure 1:**
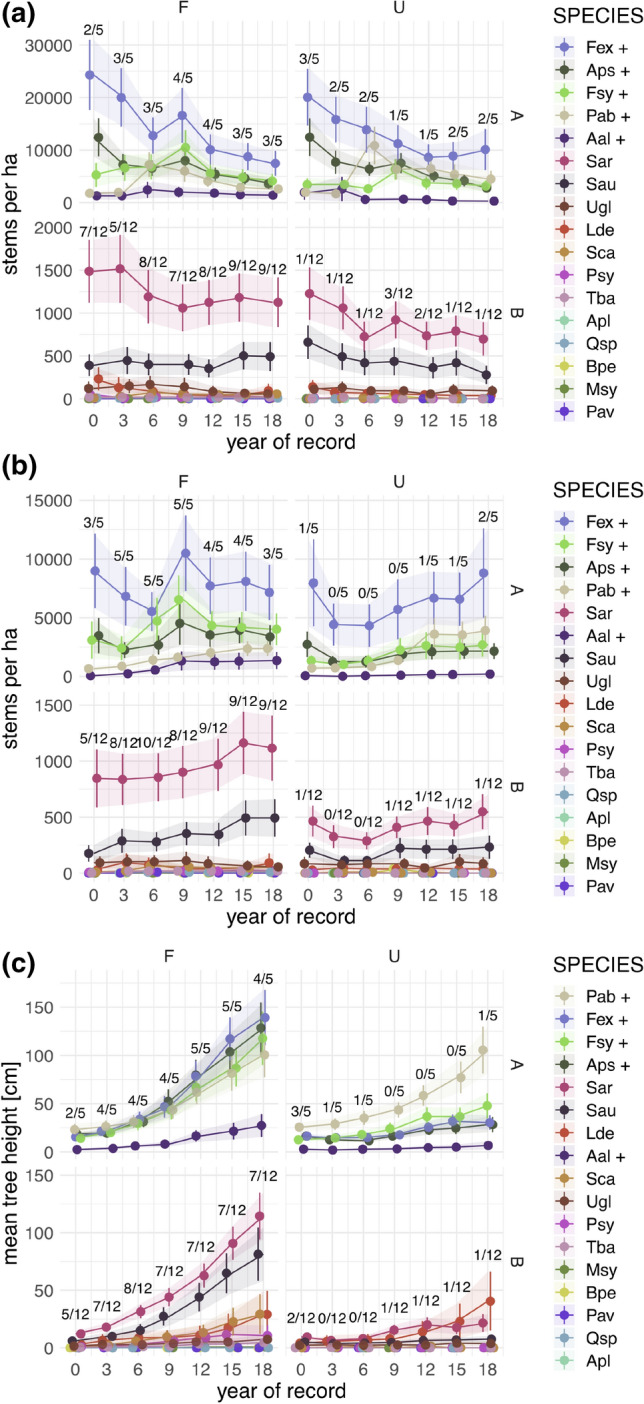
Trajectories (mean values ± std error) of (**a**) total stem density (n/ha), (**b**) stem density (n/ha) without height class one (< 10 cm) and (**c**) maximum heights per tree species for an 18-year time span, separated for the fenced plots (F) and the unfenced control plots (U); trajectories are separated for (A) the main tree species, also indicated with an + , and (B) co-occurring species. Species are ordered by their overall mean value (for total stem density, stem density without height class one and for maximums heights, respectively). Fraction numbers above the trajectories depict the number of main or co-occurring tree species on the fenced or unfenced plots, which show higher mean values (in terms of stem numbers, heights) than the remaining fraction. 3/5 at the first year of records of mean heights in panel A on the unfenced plots means, that three out of five main tree species are on average higher than the remaining two tree species. *Aal Abies alba*,* Apl Acer platanoides*,* Aps Acer pseudoplatanus*,* Bpe Betula pendula*,* Fex Fraxinus excelsior*,* Fsy Fagus sylvatica*,* Lde Larix decidua*,* Msy Malus sylvaticus*,* Pab Picea abies*,* Pav Prunus avium*,* Psy Pinus sylvestris*,* Qsp Quercus* sp.,* Sar Sorbus aria*,* Sau Sorbus aucuparia*,* Sca Salix caprea*,* Tba Taxus baccata*,* Ugl Ulmus glabra*. Please note the different, scaled y-axes in the diagrams.

Except for one tree species (i.e. rowan (*Sorbus aucuparia*) on the fenced plots) total stem numbers per hectare showed a typical overall decreasing trend in the course of the 18-year survey period (Fig. 1). At the end of the 18-year survey, ash and Norway spruce reached higher stem density on the control plots than on the fenced plots. Some rare species like Scots pine (*Pinus sylvestris*), Norway maple (*Acer platanoides*) or English yew (*Taxus baccata*) showed distinct responses to the exclosures in terms of lower maximum heights on the control plots. Within the frequently occurring tree species, European larch on average reached higher maximum heights on the control plots and Norway spruce did not respond distinctly to fencing in terms of maximum heights (within the 18-year study period; Fig. 1).

**Figure 2:**
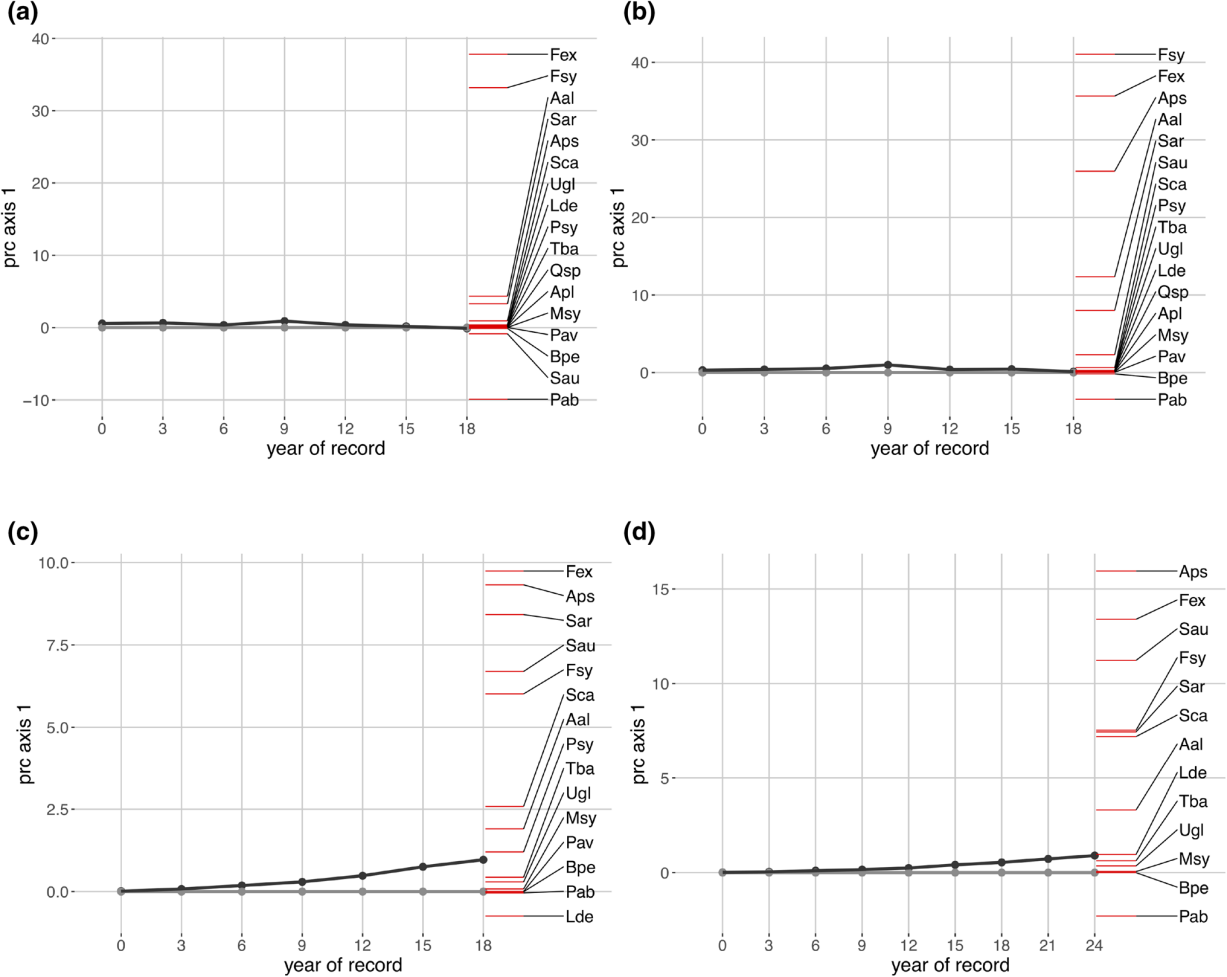
Principle response curves showing trajectories of (**a**) total stem density, (**b**) stem density without height class one (< 10 cm), (**c**) maximum heights of trees along the first RDA axis over an 18-year period and (**d**) maximum heights over a 24-year period, contrasting fenced plots (-·-·-) to open control plots ( , depicted as reference line); the second y-axis represents single species scores on the first RDA axis. Species with the same algebraic sign as the overall response curve show a corresponding response to a treatment; species with scores about zero (matching to the reference line) do not respond to the treatment or show distinctly differing response patterns; species with a opposite sign than the overall community response (-·-·-) are negatively correlated to the overall response curve. Stem number PRCs (**a**,**b**) correspond to basic stem number patterns, but fencing is non-significant in terms of permutation testing. *Aal Abies alba*,* Aps Acer pseudoplatanus*,* Bpe Betula pendula*,* Fex Fraxinus excelsior*,* Fsy Fagus sylvatica*,* Lde Larix decidua*,* Msy Malus sylvaticus*,* Pab Picea abies*,* Pav Prunus avium*,* Psy Pinus sylvestris*,* Qsp Quercus* sp.,* Sar Sorbus aria*,* Sau Sorbus aucuparia*,* Sca Salix caprea*,* Tba Taxus baccata*,* Ugl Ulmus glabra*. Please note the different, scaled y-axes in the diagrams.

These patterns were conformingly reflected by the overall PRCs with a higher (and significant) response of trees to fencing in terms of maximum heights than of stem density (Fig. 2, Table 1). Differences in stem density or maximum heights between control plots and fenced plots, expressed in species scores (Fig. 2, Table 2), corresponded to the basic observed patterns (Fig. 1) as well, e.g. with higher stem numbers of Norway spruce on the control plots (Fig. 2) or larger maximum heights of European larch on the control plots during the 18-year survey period (Fig. 2).

**Table 1:** Summary of PRCs for maximum heights (max. height), stem density including the first height class (stem no. total), and stem density without the first height class (stem no. excl. hcl 1; hcl 1 = stems < 10 cm) for the number of exclosure/control pairs (# pairs), the proportion of variance (%) captured by the time (“conditional”) and by the treatment × time interaction (“constrained”) as well as significance of permutation tests for all axes and the first axis.

Variable	Length of records	# Pairs	Proportion of variance %		Permutation significance		Year
			Conditional	Constrained	All axes	1st axis	
Max. height	9 years	82	3.9	2.8	0.001	0.001	≥ 3
	12 years	65	5.8	4.7	0.001	0.001	≥ 3
	18 years	43	7.3	6.7	0.001	0.001	≥ 6
	24 years	17	12.0	10.0	0.001	0.001	≥ 6
Stem no total	9 years	82	*1.6*	*0.3*	*0.546*	*0.449*	–
	12 years	65	*1.8*	*0.4*	*0.131*	*0.055*	–
	18 years	43	*3.0*	*0.5*	*0.329*	*0.364*	–
Stem no excl. hcl 1	9 years	82	*1.4*	*0.3*	*0.427*	*0.285*	–
	12 years	65	1.2	0.5	0.027	0.017	–
	18 years	43	0.8	0.9	0.001	0.001	–

Year indicates the point of time, when the influence of the treatment becomes significant (indicated via permutation testing; – indicates no significance at any point of time). *Non-significant PRCs* are given in *Italics.*

**Table 2:** Species scores of the tree species within the different PRCs.

Species	Species scores			
	Stem number		Maximum heights	
	18-year period		18-year period	24-year period
	Total	Without height class 1		
Pab	− 9.91	− 3.44	− 0.04	− 2.29
Aal	4.34	12.35	1.90	3.31
Lde	0.29	0.21	− 0.74	0.96
Psy	0.20	0.27	1.20	–
Tba	0.15	0.25	0.43	0.62
Aps	0.94	25.96	9.32	15.95
Apl	0.05	0.11	–	–
Fsy	33.18	41.04	6.01	7.54
Qsp	0.10	0.14	–	–
Fex	37.81	35.65	9.74	13.40
Ugl	0.34	0.22	0.29	0.35
Msy	0.01	0.04	0.08	0.06
Pav	0.01	0.02	0.00	–
Sau	− 0.86	2.29	6.69	11.23
Sar	3.30	8.01	8.42	7.45
Bpe	− 0.12	− 0.17	− 0.01	0.00
Sca	0.38	0.63	2.58	7.20

*Aal Abies alba, Apl Acer platanoides, Aps Acer pseudoplatanus, Bpe Betula pendula, Fex Fraxinus excelsior, Fsy Fagus sylvatica, Lde Larix decidua, Msy Malus sylvaticus, Pab Picea abies, Pav Prunus avium, Psy Pinus sylvestris, Qsp Quercus* sp., *Sar Sorbus aria, Sau Sorbus aucuparia, Sca Salix caprea, Tba Taxus baccata, Ugl Ulmus glabra.*

As highlighted in the summary of PRCs (Table 1), permutation tests of PRC axes did not yield significance for stem density in two thirds of tested cases. Significance for stem density was exclusively observed for stem density without height class 1 and never for total stem density. Contrary, all PRCs of maximum heights yielded significant values in permutation testing. Proportions of variance, captured by the treatment × time interaction, only reached low levels for the stem density. In contrast, all permutation tests of PRCs of maximum heights were significant and the constrained proportion of variance, reflecting treatment effects, was higher for all referring PRCs compared to stem density (Table 1). The constrained proportion of variance increased with longer duration of the observation period with highest values after 24 years. Permutation testing of the point of time, when the treatment becomes significant, only yielded results for maximum heights. Maximum heights responded to the fencing after 3–6 years. Significant deviations of the treatment plots from the control plots (= reference) were not detected at the first record (i.e. starting survey, Table 1, Fig. 2), indicating an adequate selection of comparable control and treatment plots.

Effects of ungulates on regeneration dynamics only reached comparably low levels: For the maximum heights, we observed the highest proportions of variance, captured by the treatment × time interaction (Table 1). This proportion was increasing with time. However, fencing only explained about 10% of variance of maximum heights after a survey period of 24 years. After nine years, only 3% of variance of maximum heights could be assigned to treatment × time interactions (Table 1). Contrary, time captured 12% of variance of maximum heights for the 24-year survey and 4% for the 9-year period. For stem density, all referring (significant) values were much lower.

### Sensitivity of PRC to sample size

To test for the sensitivity of our PRCs to sample size, we also ran PRCs with randomly reduced data sets (Tables 3 and 4). Thereby, we used the number of exclosure/control pairs, which were available for the longer lasting survey periods (i.e. 65, 43, or 17 plot pairs). We further performed a random sampling with replacement building subsets for the first two surveys (82 and 65 pairs of sampling plots respectively) consisting of 82 (for the first survey only), 65, 43 and 17 pairs of sampling plots with 500 iterations each.

**Table 3:** Summary of PRCs for maximum heights (max. height) for the original number of exclosure/control pairs (# pairs) of the 9-year and 12-year period and for randomly selected reduced numbers of exclosure/control pairs.

Length of records	# Pairs	Proportion of variance %		Permutation significance		Year
		Conditional	Constrained	All axes	1st axis	
9 years	82	3.8	2.8	0.001	0.001	≥ 3
	65	3.9	2.8	0.001	0.001	≥ 6
	43	4.0	3.4	0.001	0.001	≥ 6
	17	4.1	3.7	0.001	0.001	–
12 years	65	5.8	4.7	0.001	0.001	≥ 3
	43	6.2	5.2	0.001	0.001	≥ 6
	17	5.5	5.1	0.001	0.001	–

The proportion of variance (%) captured by the time (“conditional”) and by the treatment × time interaction (“constrained”) as well as significance of permutation tests for all axes and the 1^st^ axis are given. Year indicates the point of time, when the influence of the treatment becomes significant (indicated via permutation testing; –- indicates no significance at any point of time). Non-significant PRCs in terms of permutation testing are given in *Italics.*

**Table 4:** Summary of PRCs for maximum heights (max. height) for the original number of exclosure/control pairs (# pairs) of the 9-year and 12-year period and for bootstrapped, reduced numbers of exclosure/control pairs.

Length of records	# Pairs	Proportion of variance (%) original data		Proportion of variance (%)bootstrap sample x ± sd	
		Conditional	Constrained	Conditional	Constrained
9 years	82	3.8	2.8	3.9 ± 0.6	3.2 ± 0.7
	65	●	●	4.1 ± 0.7	3.3 ± 0.8
	43	●	●	4.1 ± 0.8	3.5 ± 1.0
	17	●	●	4.6 ± 1.4	4.5 ± 1.7
12 years	65	5.8	4.7	6.0 ± 0.9	5.1 ± 0.9
	43	●	●	6.2 ± 1.0	5.3 ± 1.2
	17	●	●	7.0 ± 1.7	6.6 ± 2.0

The proportion of variance (%) captured by the time (“conditional”) and by the treatment × time interaction (“constrained”) are given for the original data, the mean values ± standard deviation are given for the bootstrap samples.

Except for one case, proportions of variance captured by the treatment × time interaction were not distinctly higher for the reduced data sets (Tables 3 and 4).

### Tree species response

In total, we observed 19 tree species on the survey plots (Table 5). Of these, some species appeared after the first survey, some species like Scots pine (*Pinus sylvestris*) or wild cherry (*Prunus avium*) disappeared in the course of the experiment and some species could only be recorded in low numbers on single plots (*Tilia* sp., *A. campestris*, *Quercus* sp.).

**Table 5:** Ranking of tree species (maximum heights) in terms of their affinity to the referring first PRC axis; affinity.

Years	# Pairs	*Picea abies*	*Larix decidua*	*Acer pseudoplatanus*	*Fagus sylvatica*	*Fraxinus excelsior*	*Salix caprea*	*Sorbus aria*	*S. aucuparia*	*Abies alba*	*Pinus sylvestris*	*A. platanoides*	*Betula pendula*	*Malus sylvestris*	*Ulmus glabra*	*Taxus baccata*	*Prunus avium*	*Tilia sp.*	*A. campestris*	*Quercus sp.*
9	83	0	+	+ +	+ +	+ +	+ +	+ +	+ +	+	0	0	0	0	0	0	0	0	0	0
12	65	0	0	+ +	+ +	+ +	+ +	+ +	+ +	+	+	0	0	0	0	0	0	0	●	●
18	43	0	-	+ +	+ +	+ +	+ +	+ +	+ +	+ +	+ +	0	0	0	0	0	0	0	●	●
24	17	–	+	+ +	+ +	+ +	+ +	+ +	+ +	+ +	●	0	0	0	0	0	●	●	●	●

++ high affinity, + moderate affinity, 0 no corresponding response,— opposite response; ● not occurring or very rare.

For the 24-year period, sycamore maple showed the most distinct positive response to fencing in terms of maximum heights, followed by ash and rowan (*Sorbus aucuparia*) (Fig. 2, Table 5). Furthermore, European beech, common whitebeam (*Sorbus aria*) and goat willow (*Salix caprea*) showed high affinity to the first axis of the principle response curves. Maximum heights of silver fir followed a similar pattern. Several species like English yew, wych elm (*Ulmus glabra*), European crab apple (*Malus sylvestris*), Norway maple (*Acer platanoides*) and silver birch (*Betula pendula*) only reached species scores around zero, indicating a weak affinity to the first axis of the principle response curves. In contrast, Norway spruce was the only tree species following an opposite pattern of heights of highest trees, with higher trees on the control plots at the end of the 24-year survey period. However, the low proportion of variance explained by the treatment (10%) should be considered.

### Traditional approach (LM) vs. PRC—contrasts at tree species level

The LMs of the maximum height values per tree species as response variables and the treatment, the point of time and their interaction as fixed factors yielded adjusted R^2^ values between zero (for silver birch, European larch, European crab apple, wild cherry, Scots pine) and a maximum of 24% (for common whitebeam) (see Table 6). Seven out of 15 species-specific maximum height models comprised significant interactions of the treatment and the point of time. No single species-specific LM yielded a significant coefficient of the main factor treatment, whereas three models (i.e. for silver birch, European larch and Norway spruce) yielded a significant effect of point of time without comprising significant interactions between the treatment and the point of time. Contrasting the species-specific maximum height models to the partial species-specific results of the PRC showed, that most models go conform with the PRC in terms of significance and algebraic sign of the effect coefficients of the LMs (Table 6).

**Table 6:** LMs of tree species´ responses in terms of recruitment/maximum heights (height_max_spec_ ~ time + treatment + time:treatment) vs. partial (species-specific) results of the PRC.

LMs																PRC	Comparison LM vs. PRC
Tree species	Estimates														Ad-justed R^2^	Affinity of tree species to the related 1st PRC axis	Con-formity of results
	Inter-cept	Time						Treatment	Interaction								
		Year 3	Year 6	Year 9	Year 12	Year 15	Year 18	Fence	Year 3: fence	Year 6: fence	Year 9: fence	Year 12: fence	Year 15: fence	Year 18: fence			
Aal	2.95	− 0.95	− 0.05	0.30	1.51	2.02	3.84	− 0.42	2.09	3.72	5.35	12.56	17.02	**21.05**	0.04	+ +	+
Aps	16.58	− 3.93	− 4.91	− 0.07	6.09	8.14	11.72	2.16	4.63	17.49	33.70	**54.70**	**76.72**	**98.02**	0.18	+ +	+ +
Bpe	0.00	0.00	0.00	**0.42**	0.00	0.00	0.00	0.00	0.00	0.00	− 0.42	0.00	0.00	0.00	0.00	0	+ +
Fex	16.44	− 1.65	− 1.51	1.28	9.19	15.30	14.02	− 0.86	7.54	19.81	29.93	**53.70**	**86.21**	**109.51**	0.19	+ +	+ +
Fsy	12.67	1.79	5.44	11.35	23.98	24.00	**35.16**	1.44	4.09	9.88	17.61	28.86	**48.65**	**68.33**	0.11	+ +	+ +
Lde	4.02	1.63	2.63	3.84	9.77	18.84	**36.51**	− 1.40	2.26	3.47	2.56	− 1.47	− 4.02	− 10.07	0.00	−	+
Msy	0.00	0.00	0.00	0.00	0.00	0.00	0.00	0.00	0.00	0.00	0.00	0.77	0.77	0.77	0.00	0	+ +
Pab	**25.74**	3.00	9.44	17.77	32.72	**51.28**	**79.81**	− 2.63	0.35	− 1.21	2.63	5.77	7.07	− 2.35	0.08	0	+
Pav	0.00	0.00	0.00	0.00	0.00	0.00	0.00	0.00	0.00	0.42	0.00	0.00	0.00	0.00	0.00	0	+ +
Psy	0.12	0.00	− 0.12	− 0.12	− 0.12	− 0.12	− 0.12	0.77	0.40	2.33	4.88	8.07	10.74	9.93	0.00	+ +	−
Sar	9.49	− 3.07	− 1.49	5.88	10.54	8.19	12.14	2.61	8.93	20.79	26.14	**40.14**	**70.51**	**90.30**	0.24	+ +	+ +
Sau	5.12	− 1.44	− 0.91	− 0.37	1.30	1.72	2.40	0.91	5.33	9.93	21.67	**36.81**	**57.14**	**72.93**	0.13	+ +	+ +
Sca	0.00	0.42	1.28	0.00	1.40	0.00	0.00	0.42	1.33	4.35	9.19	11.44	22.26	**28.88**	0.03	+ +	+
Tba	0.00	0.23	0.00	0.00	0.00	0.00	0.00	0.23	0.37	0.95	1.30	3.02	3.02	4.42	0.01	0	+ +
Ugl	2.28	0.30	− 0.05	0.26	0.19	1.74	1.40	− 0.67	0.98	1.16	1.81	3.4	1.884	4.33	− 0.01	0	+ +

Conformity of results of LMs with the related 1st PRC axis for example is high (+ + or +), when significant time:treatment interaction terms of the LM of a species correspond to a high affinity of the same species to a significant time:treatment principle response curve and vice versa.

*Aal Abies alba, Aps Acer pseudoplatanus, Bpe Betula pendula, Fex Fraxinus excelsior, Fsy Fagus sylvatica, Lde Larix decidua, Msy Malus sylvaticus, Pab Picea abies, Pav Prunus avium, Psy Pinus sylvestris, Qsp Quercus sp., Sar Sorbus aria, Sau Sorbus aucuparia, Sca Salix caprea, Tba Taxus baccata, Ugl Ulmus glabra.*

## Discussion

### Analysing herbivore effects on the height of largest trees versus total stem density

As only a comparatively small number of progenies of forest trees survive till maturity^74^ and only a small portion of seedlings finally grows into the forest canopy^38^, we hypothesised that dynamic recording of heights of the highest tree seedlings, saplings and young trees per tree species (and plot) to be more meaningful than taking total stem density (see^30^) to depict net effects of herbivory on forest dynamics at relevant developmental stages (i.e. terminal heights being no longer accessible for browsing by ungulates). This hypothesis was confirmed through trajectories of maximum heights per tree species that yielded significant results of PRCs contrary to trajectories of total stem density. More significant responses of tree communities to browsing in terms of height growth than in total stem numbers have already been documented in previous studies^30^. Since the highest trees have a higher likelihood of canopy recruitment as compared to average individuals^42,43^, an analysis of average responses only leads to a misinterpretation of herbivore effects^41,75,76^. A dynamic recording of highest trees that allows for shifting dominance focuses on net results of regeneration processes and includes compensation of losses in height growth caused by browsing by other, non-browsed tree individuals at community level^41,74^. For monitoring of ungulate impacts in practical forestry and wildlife management, the focus on highest trees also has advantages in terms of lower recording efforts in the field.

We assumed that trajectories of height growth of all occurring trees of each species might be less relevant than height dynamics of the respective highest trees of each species, as herbivory-mediated suppression of one tree might favour height growth of a neighbouring tree (even of the same species) and this spatial impact might change with time.

This is in line with results of other studies^77^, predicting selective effects of browsing in terms of reduced abundance of small trees, which in turn results in a lower recruitment into higher height classes. Modelling juvenile survivorship of tree species, mortality probabilities of saplings might be predicted as functions of recent growth histories^42^. Height class distributions of plant taxa or functional groups are typical response variables when addressing herbivore effects on plant communities (see^77^). However, for tree species composition of mature forests the number and mixture of trees in the reproductive upper layer is important. In contrast to the high numbers of trees in forest regenerations with initial seedling numbers up to some 100,000 trees per hectare, in mature forests, the tree density naturally decreases to some hundred trees per hectare. For this decrease, many causes are possible (compensatory mortality). Within this forest dynamic, the number of surviving trees and the heights of the species’ dominant trees in each stage of forest development are decisive whereas total seedling densities and their height classes are less important for the further forest development (cf.^41,43,51,78^). In agreement with other authors^79^, we see deer browsing on woody seedlings as a function of species preference, height structure, growth, and seasonal phenology of seedlings. Maximum height dynamics, addressed in our study, reflect local dominance of tree species via asymmetric plant competition, and such effects, both within and among forest patches, can accrue over time shaping forest structure and composition. As in other studies, high variance in growth rates might be attributed to differences in phenotypes, resulting from various impacts like canopy shading, variation in soil nutrient and moisture conditions, herbivory, or intraspecific competition^76,80^. Accordingly, growth rates can vary widely even under controlled experimental conditions^81^.

### Community approach versus single-species focus

In previous studies, most species-oriented approaches either focused on single (often rare or dominant) target species or were based on community-related values (e.g. diversity indices; see^82–84^). As regeneration dynamics and survivorship of tree species unfold as a result of neighbourhood processes of individuals and their species-specific life-history traits in connection with environmental filters^29,35,85–88^, a synchronous analysis of both the entire regenerating tree community and all tree species (vs. subjectively focusing on certain species only) seems to be essential. However, many standard statistical approaches like GL(M)Ms are not applicable in case of largely multiple response variables. Such approaches are typically applied to total stem numbers, to regeneration cover or to responses of single key species (e.g.^89^). In contrast, PRCs proved to yield traceable results at community *and* species level. For species with weak affinity to the first PRC axis, pictures of the second and following axis might be relevant^57^. Thereby, proportions of variance explained by the treatment × time interaction should be considered.

### Long term versus short term studies

The proportion of variance in maximum heights, explained by the treatment × time interaction (i.e. the fencing) was 3% after nine years but increased to about 10% after 24 years of monitoring. This highlights the importance of long-term surveys, when exploring herbivore effects on forest regeneration (see also^30^) and confirms our second hypothesis. In half of our analyses, PRCs became significant at the 6th year of monitoring and at subsequent phases. We thus infer, that short-time surveys might not catch relevant developmental stages (i.e. heights of terminal shoots being no longer accessible for browsing ungulates) and might thus not adequately represent net effects of browsing at community level. Even at tree species level, response patterns to herbivory might not be uniform over time: For example, both Norway spruce and European larch changed in their response to fencing. Norway spruce showed no distinct response in terms of maximum heights over 18 years and negatively responded to fencing afterwards. Contrary, European larch switched from positive response to neutral and negative response to fencing and ended after 24 years with positive response in terms of maximum heights. Specific responses of tree species to ungulate browsing might derive from different feeding preferences of ungulates, selecting for the most palatable species and from tree species´ tolerance to browsing^52,90,91^. Most likely, the observed patterns for Norway spruce and European larch reflect their low preference by browsing ungulates^92^ due to low palatability and effects of competition with broadleaf trees on the fenced plots. Furthermore, European larch typically germinates successfully on exposed mineral soil, which might be promoted by ungulate trampling (see^93^). Windows of opportunity for successful recruitment of European larch might thus positively correlate with periods of higher ungulate presence.

The percentages of variance explained by the PRCs appear to be rather low. It is thus evident that, despite considerable population densities of ungulates, other factors than browsing distinctly drove the dynamics of the largest individuals per species (and plot) in our study such as responses to (micro-)sites and light conditions, intra- and interspecific competition and pathogens^29,35,85–88^. We attribute this to the following causes: (1) In the montane forests of the study region, the natural species composition has a high ratio of Norway spruce^49^, and the ratio has been further increased by forestry since the mid of the nineteenth century^94,95^. The response of Norway spruce to the fencing was weak and, at the end of the survey period, contrary to all other tree species. The low percentages of variance explained may thus carry a strong signal of this species; (2) The study sites represent mesic conditions on average and did not show strong disturbances during the observation periods. On such sites, herbivory-caused mortality or retarding of individual growth through herbivory may be compensated at community or individual levels. This may not be the case on e.g. drier sites after large disturbances in comparable regions, where intense ungulate herbivory had strong effects on post-disturbance species composition and lead to higher soil CO_2_-effluxes caused by higher soil temperatures^96^. Moderate intensities of herbivory, however, did not show differences in soil CO_2_-effluxes^96^. It may thus be that thresholds exist in terms of impacts of ungulate herbivory on ecosystem parameters that were not crossed on the sites in our study area.

In the given study, we focused on effects of ungulate herbivory at different developmental stages of tree species, targeting their chance of reaching reproductive size. Future analyses might as well address bottom-up forces (abiotic factors) together with top–down effects as related data have been recorded on the study plots. Other factors, additionally affecting forest regeneration structures and partly interacting with ungulate impacts, could be included in further studies testing different constellations for long-term effects on forests, e.g. changing light conditions in the understorey, other abiotic site factors, frequency of seed trees per species, insects, rodents, tree diseases, competitive vegetation such as shrubs, grasses, and herbs.

### General considerations for analyses and interpretations of exclosure experiments

For analyses and interpretations of exclosure experiments—as done in this study—several factors have to be considered: (1) tree regeneration in control plots and fenced plots should not differ at the beginning of records. In other words, the status of already present regeneration, site conditions and the presumed development of the crown canopy should be the same for both plot types. Thus, the choice of treatment–control pairs is a crucial point in sampling design. In our case, we did not find any significant deviations of the treatment (fenced) plots from the control plots (= reference) for the starting survey. We thus conclude, that we adequately selected comparable control and treatment plots. (2) Due to the sampling design, control-fence plots are usually located in areas with beginning/already existing forest regeneration. In case that the surroundings of small regeneration spots are comparatively poor in forest regeneration (and ground vegetation) cover (depending on site and silvicultural system), regeneration at such small spots might be more intensively used by ungulates and herbivory effects might be boosted there, compared to regeneration on extended areas. In our study, the high number of control-fence pairs should compensate for such possibly occurring effects. Furthermore, PRCs proved to be comparatively non-sensitive to changes in sample size. Potential compensation effects or amplification effects of deer impacts on forests trees might occur at later stages (older thickets, pole stand). Such effects could not be investigated in the given study up to now (needing longer periods than 24 years), but the exclosures hold the potential for referring future analyses.

## Conclusion

Trees show a strong ontogenetic variation in terms of consumption rates by herbivores. Effects of herbivory, occurring at early developmental stages, might be accompanied or followed by a cascade of other processes, that drive plant community dynamics. Using principle response curves (PRC) we could show, that early stage herbivory might thus not be a significant driver of dynamics at later stages and it might not necessarily determine the chance of reaching reproductive size in forest trees. Repeated records of the heights of the highest individuals per tree species captures changes in dominance with time, focusing on individuals with a high probability of reaching the main canopy and getting into reproductive stages. Recording maximum heights of all regenerating tree species in paired plots with and without exclosures thus better reflects effects of ungulate herbivory on long-term dynamics of tree regeneration than recording stem densities. Results from surveys with short and medium duration differ from long-term results, when trees have reached later development stages (thickets, pole stands). Regeneration dynamics and survivorship of tree species are the result of neighbourhood processes of individuals and species-specific life-history traits together with environmental filters. Thus, synchronous analyses of both the entire regenerating tree community and all occurring tree species (vs. subjectively focusing on certain target species) are ecologically informative.

## Data Availability

Datasets of the enclosure-control pairs are available at https://doi.org/10.6084/m9.figshare.13160159.v1.

## Code availability

Program source codes are maintained by U. Nopp-Mayr.
